# Perioperative cytokine profile during lung surgery predicts patients at risk for postoperative complications—A prospective, clinical study

**DOI:** 10.1371/journal.pone.0199807

**Published:** 2018-07-03

**Authors:** Kai B. Kaufmann, Sebastian Heinrich, Hans Felix Staehle, Lioudmila Bogatyreva, Hartmut Buerkle, Ulrich Goebel

**Affiliations:** 1 Department of Anesthesiology and Critical Care, Medical Center–University of Freiburg, Faculty of Medicine, University of Freiburg, Freiburg, Germany; 2 IMBI, Institute of Medical Biometry and Statistics, University of Freiburg, Freiburg, Germany; Catalan Institute of Oncology, SPAIN

## Abstract

**Background:**

Postoperative complications after lung surgery are frequent, having a detrimental effect on patients’ further course. Complications may lead to an increased length of hospital stay and cause additional costs. Several risk factors have been identified but it is still difficult to predict contemporary which patients are at risk. We hypothesized that patients who show an increased inflammatory response at the time of wound closure and 24 hours after surgery are at risk of postoperative complications within 30 days after surgery.

**Methods:**

Postoperative complications (pulmonary, cardiac, neurological and renal) of 96 patients scheduled for lung surgery at the Medical Center–University of Freiburg were analyzed in this prospective, clinical study. Blood samples for cytokine analysis (Interleukin (IL)-6, IL-8, IL-10, Tumor necrosis factor [TNF]-α, IL-1ß and IL12p70) were taken before surgery, at wound closure and 24 hours after surgery. Cytokine levels of patients with and without postoperative complications were analyzed by Receiver operating characteristic (ROC) curve analysis. To adjust the results according to existing covariates a multivariate logistic regression analysis was conducted.

**Results:**

The complication and non-complication group differed significantly according to nicotine dependency, Angiotensin-receptor-II blocker medication, rate of thoracotomy and preoperative lung function. The intraoperative hemodynamic parameters and therapy did not differ between the groups. Twenty-nine patients (30%) developed postoperative complications within 30 days after surgery. Plasma concentrations of IL-6, IL-10 and IL-8 at the time of wound closure and 24 hours after surgery were higher in the complication group. Multivariate regression analysis on postoperative complications revealed an Odds ratio of 56 for patients with IL-6 and IL-8 levels above the 3^rd^ quartile measured on the first postoperative day.

**Conclusions:**

Perioperative detection of increased plasma concentrations of inflammatory cytokines in lung surgery may be used in addition to other clinical predictors to identify patients at risk for postoperative complications.

**Trial registration:**

German Clinical Trials Register 00006961.

## Introduction

Postoperative complications after lung surgery, particularly of pulmonary origin, are the main reason for a prolonged hospital stay with an incidence of up to 24%.[1] Apart from pulmonary complications patients undergoing lung surgery also suffer from cardiac, renal and neurological complications with incidences of up to 11%, 7% and 2%, respectively.[2–4] The number of patients undergoing lung cancer surgery will further increase as it still is the only curative therapy.[5] Several studies have assessed patients’ risk for postoperative complications after thoracic surgery. Major risk factors identified in these studies were American Society of Anesthesiologists (ASA) score ≥ 3, decreased forced expiratory volume in 1 second (FEV_1_) preoperatively, smoking, age and thoracotomy whereas predictive biomarkers have not been taken into consideration.[2,4,6] Non-cardiac thoracic surgery leads to a systemic release of pro- and anti-inflammatory cytokines especially on the day of surgery and first postoperative day.[7–9] Apart from the trauma caused by surgical incision, patients’ lungs experience ischemia/reperfusion injury due to one-lung ventilation.[10] As a consequence, macrophages in the alveoli secret a variety of cytokines. In patients with postoperative pulmonary complications after major abdominal surgery, levels of Interleukin (IL)-6 and IL-8 were significantly increased on the first postoperative day. However, none of the ILs reached a sufficient prognostic value.[11] Patients with pulmonary complications after esophagectomy showed increased levels of IL-6 and IL-10.[12] IL-6 was used as a predictive biomarker of systemic inflammatory response after non-cardiac thoracic surgery without analyzing a possible impact on postoperative complications.[7] To date, one study has focused on lung surgery comparing postoperative inflammation and outcome; IL-6 plasma concentrations of patients suffering from postoperative complications did not differ from those without complications.[13] As far as we know, there is no published data showing an early difference in the cytokine expression of lung surgery patients predicting postoperative complications. In this study, we hypothesized that inflammatory cytokines measured at the time of wound closure and on the first postoperative day can identify patients at risk of postoperative complications after lung surgery.

## Methods

This prospective, clinical trial to evaluate inflammatory cytokines’ predictive character for postoperative complications is a pre-planned sub-study of a randomized clinical two-arm trial approved by the local Ethics Committee Freiburg, Germany [EK 502/14] on 16^th^ of December 2014 and was registered in the German Clinical Trials Register (DRKS No. 00006961) on the 2^nd^ of February 2015 (S1 File).[14] This trial was conducted at the Department of Anesthesiology and Intensive Care and the Department of Thoracic Surgery, Medical Center–University of Freiburg, Faculty of Medicine, University of Freiburg, Germany (S2 File).

### Objective

The aim of this study was to compare plasma concentrations of different inflammatory cytokines at an early stage after lung surgery and to validate these biomarkers’ predictive character for postoperative complications. In contrast to the initial study focusing on goal-directed therapy, group division into two groups was based on general postoperative complications as a composite outcome due to the fact that postoperative complications are linked to each other. The occurrence of one postoperative complication affecting one organ predisposes for further organ dysfunction. Although multifactorial, we consider the early inflammatory response after surgery to be the general origin of postoperative complications. Due to the small sample size and high interindividual variability in cytokine response focusing on only one type of complication would not be valuable. As a consequence, with regards to the increase of postoperative inflammatory cytokines we focused on all complications. The main focus of this pilot study was to identify patients at risk for postoperative complications as early as possible (at the end of surgery/ 24 hours after surgery) to prevent these by taking those patients into a preventive program with an extended respiratory therapy or increased attention on postoperative goal-directed therapy, for example. With the model used in this trial we are not able to anticipate the exact onset of postoperative complications. In this study all postoperative complications that occurred within 30 days after surgery were recorded without focusing on the exact time of onset. However, this trial was meant as a pilot study showing that in addition to known clinical risk factors, inflammatory biomarkers can be used to identify patients at risk at an early stage to act preemptively.

### Patients

**Fig 1:**
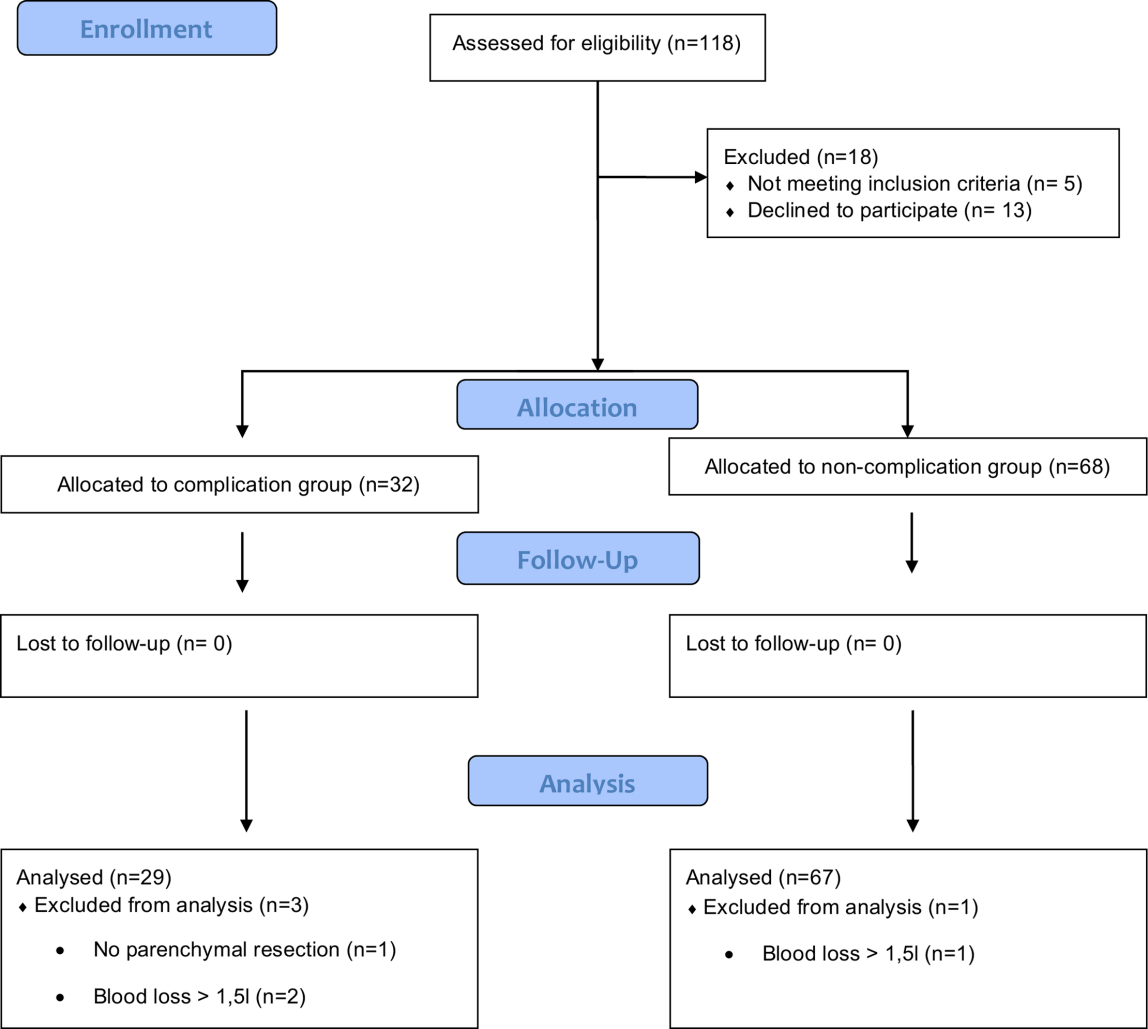
CONSORT flow chart.

Written informed consent was obtained for participation in the research discussed in this manuscript from every patient enrolled in this study. The authorization to draw blood was included a priori in the written informed consent obtained from all patients enrolled in the randomized controlled parallel-arm trial. The authors confirm that all ongoing and related trials for this intervention are registered. Patients were enrolled and followed-up from February 2^nd^ 2015 until June 30^th^ 2015. The follow-up for postoperative complications was 30 days. One hundred and eighteen patients were considered eligible for this study (Fig 1). Inclusion criterion was lung parenchyma due to lung cancer. Several exclusion criteria were defined. These were automated implantable cardioverter defibrillator or pacemaker, age < 18 years, emergency surgery, New York Heart Association Functional Classification 4, morbid obesity (body mass index [BMI] > 50 kg m^-2^), esophageal pathologies, intraoperative blood loss of more than 1.5 liter, pregnancy, intraoperative use of diuretics, cardiac valve pathologies and intraoperative blood transfusion. Final data analysis was conducted on 96 patients (Fig 1). All patients received a spirometric analysis prior to the operation.

### Cytokine analysis

Blood samples were taken before induction of anesthesia, at wound closure and 24 hours postoperatively. Samples were centrifuged at 1500 rounds per minute for 10 minutes. The supernatant was used for cytokine analysis and stored at -80°C. For cytokine analysis, the BD cytometric bead array human inflammatory cytokines kit [Becton Dickinson, Heidelberg, Germany, cat. #551811] with the corresponding software for flow cytometry was used according to the instruction manual. The following cytokines were measured: IL-6, IL-8, IL-10, Tumor necrosis factor [TNF]-α, IL-1ß and IL12p70.

### Anesthetic protocol

Patients received an epidural catheter (thoracotomy) or a paravertebral catheter (video-assisted thoracoscopy) just before anesthesia induction. A total of 10 ml of ropivacaine 0.2% and sufentanil 0.2 μg/kg was injected before start of surgery via epidural catheter, 10 ml of ropivacaine 0.2% was used for the paravertebral catheter and sufficient analgesia was achieved by a continuous infusion of ropivacaine 0.2% at 8 ml/h during surgery. Both types of catheters were used until the 5^th^ postoperative day. In every patient enrolled in the study continuous blood pressure was monitored by radial artery cannulation. Central venous access was only established if patients underwent thoracotomy or were scheduled for extended thoracoscopic lung resections. Anesthesia was induced by intravenous sufentanil 0.5μg/kg and target-controlled infusion of propofol achieving plasma concentrations of 2.5–4 μg/ml according to an adequate depth of anesthesia measured by bispectral index of the electroencephalography. A bispectral index of 40 to 60 was considered adequate. For endotracheal intubation with a double-lumen tube and during the operation cis-atracurium (0.1 mg/kg bodyweight [BW]) was used. During surgery depth of muscle relaxation was monitored continuously. For all patients of the study lung-protective ventilator settings were applied. Under pressure-controlled ventilator settings positive endexspiratory pressure was set to 5 cm H_2_O and peak pressure of less than 30 cm H_2_O was kept for double-lung and one-lung ventilation. The inspired oxygen fraction was kept as low as possible to reach a peripheral oxygen saturation higher than 90%. During one-lung ventilation the tidal volume was kept at 6 ml/kg BW with a respiratory rate of 10 to 15/min. At the end of one-lung ventilation sufficient re-inflation was achieved by a manual recruitment maneuver for 10 seconds to 30 cm H_2_O five times. All patients were successfully extubated in the operating room and transferred to the intermediate care unit.

### Postoperative complications

Postoperative complications were recorded within 30 days after surgery and defined as reported previously: pulmonary complications including atelectasis, pneumonia, pleural empyema, respiratory failure, pulmonary embolism and bronchopleural fistula that required reoperation. [6,14,15] Cardiac and renal complications included myocardial infarction, new onset of atrial fibrillation and acute kidney injury according to the Acute Kidney Injury Network criteria.[15] Neurological complications included stroke and delirium.[15] All outcome parameters were documented by a research personnel who was not involved in patients’ treatment.

### Statistical analysis

The primary outcome of the initial randomized-controlled trial was the incidence of postoperative pulmonary complications (PPCs). [14] Therefore the sample size calculation was based on a previously reported incidence of PPCs after lung surgery of approximately 24%.[1] We aimed to detect a reduction in the postoperative pulmonary complication rate to 3% with a power of 80% and a two-sided significance level of 5%, the calculated sample size was 48 patients per group. [14] Sample size calculation based on cytokine levels of patients with general postoperative complications after lung resection surgery is difficult to assess because data on these suspected cytokine levels are scarce and of high interindividual variability. To the best of our knowledge there is just one study that focused on lung surgery investigating PPCs and postoperative inflammation. [13] Studies focusing on lung surgery, postoperative general complications and cytokine levels are pending.

Categorical data were analyzed using Fisher’s exact test. Continuous variables were examined for normal distribution. If the data were normally distributed, independent samples t-test for normally distributed variables was performed. In case of not normally distributed data, the Mann-Whitney U test was used. To test the prognostic capability of interleukin plasma concentrations to distinguish between patients who will develop postoperative complications and those who will not, the receiver operating characteristic (ROC) curve analysis was performed. To investigate the best cut-off value that distinguishes between patients with and without complications the Youden Index was calculated. Due to the imbalance of baseline factors which needs an adjusted prediction model by implementing existing covariates, a multiple regression analysis was conducted. Statistical analysis was performed using IBM SPSS Statistics 22 software.

## Results

One-hundred and eighteen patients were assessed for eligibility. Hundred patients were assigned. Of the 18 patients not included in the study, five did not meet the inclusion criteria, whereas of 13 patients formal informed consent was not obtained. Four patients who were initially allocated were excluded, three patients because of a blood loss of more than 1.5 liters intraoperatively and one due to intraoperative finding of a neuronal tumor not requiring lung parenchyma resection. Ninety-six patients were finally analyzed (Fig 1).

Twenty-nine (30%) patients developed postoperative complications within 30 days after surgery (Table 1). The total number of complications varies from the number of patients with postoperative complications as some patients had more than one complication. There were four of these. One patient showed three complications, whereas three patients suffered from two complications. Without double-counting there were 29 patients with postoperative complications. The initial group allocation of the randomized-controlled trial into Esophageal-doppler monitoring (EDM) Goal-directed therapy (GDT) versus Control did not reveal significant differences according to the number of patients with postoperative complications (S1 Table). Analysis of overall complications (pulmonary, cardiac, renal and neurological) showed a significant difference between the EDM GDT and Control group (11 vs. 23, *P* = 0.018) (S1 Table). However, this difference is due to the difference in PPCs between the groups. Focusing on postoperative complications excluding PPCs there is no difference between the EDM GDT and control group. Apart from that, there were patients who showed more than one complication, actually there were four of these. Without double-counting there were 11 patients with postoperative complications in the EDM GDT group and 18 patients in the Control group (*P* = 0.13) (S1 Table). We considered it to be more accurate to compare total amount of patients with complications as postoperative complications are linked to each other, i.e. developing one complication predisposes for further postoperative complications. [16] According to current literature we presume that the early inflammatory response after surgery play a central role in the origin of postoperative complications so that the comparison of total amount of patients with complications which showed no difference between the treating arms of the initial study appears accurate. [8,11–13] Regarding the overall number of postoperative complications atelectasis was the most frequent one with 10 (10%), followed by pneumonia 6 (6%), pleural empyema 2 (2%), respiratory failure 2 (2%), pulmonary embolism 2 (2%), Broncho-pleural fistula 1 (1%), acute kidney injury 3 (3%), myocardial infarction 2 (2%), atrial fibrillation 4 (4%), stroke 1 (1%) and delirium 1 (1%) (Table 1).

**Table 1:** Postoperative complications.

Atelectasis	10 (10%)
Pneumonia	6 (6%)
Pleural empyema	2 (2%)
Respiratory failure	2 (2%)
Pulmonary embolism	2 (2%)
Broncho-pleural fistula	1 (1%)
Acute Kidney Injury	3 (3%)
Myocardial infarction	2 (2%)
Atrial fibrillation	4 (4%)
Stroke	1 (1%)
Delirium	1 (1%)
Total complications	34 (35%)
Total amount of patients with postoperative complications	29 (30%)

Definition according to the **E**uropean **P**erioperative **C**linical **O**utcome definitions (modified [15]) Data are presented as number of patients and percentage of all analyzed patients.

### Baseline patient and surgical characteristics

Of all baseline characteristics (Table 2) the following parameters differed significantly between patients with and without postoperative complications: continued nicotine dependency, angiotensin II receptor blockers (ARBs), surgical access (thoracoscopy versus (vs.) thoracotomy) and preoperative respiratory function. Twenty-three (79%) patients in the postoperative complications group showed a continued nicotine dependency compared to 38 (57%) patients in the non-complication group (*P* = 0.04). One patient (3%) in the complication group was on ARBs therapy whereas 14 (21%) patients in the non-complication had this medication (*P* = 0.03). In the complication group four patients (14%) underwent thoracoscopy and 25 (86%) thoracotomy whereas in the non-complication group 28 patients (42%) underwent thoracoscopy and 39 patients (58%) had a thoracotomy (*P* = 0.009). Considering the preoperative respiratory function patients in the complication group had a preoperative FEV_1_ 68% (interquartile range: 54–86%), vital capacity (VC) 85% (interquartile range: 72–104%) and peak expiratory flow (PEF) 69% (interquartile range: 52–82%) compared to the non-complication group with FEV_1_ 80% (interquartile range: 66–98%), VC 99% (interquartile range: 87–111%) and PEF 77% (interquartile range: 61–96%) (*P* = 0.008; *P* = 0.013 and *P* = 0.05).

**Table 2:** Patient and surgical characteristics.

		Complications	No complications	*P* value
		(n = 29)	(n = 67)	
Age [years]		65 (60–73)	64 (51–73)	0.3
BMI [kg/m^2^]		26 (23–29)	26 (23–29)	0.9
Sex [n]	male	18 (62%)	35 (52%)	0.5
	female	11 (38%)	32 (48%)	0.5
ASA [n]	1	0	0	
	2	3 (10%)	11 (17%)	0.54
	3	24 (83%)	53 (80%)	0.78
	4	2 (7%)	2 (3%)	0.58
Comorbidity [n]				
	Nicotine dependence	23 (79%)	38 (57%)	**0.04**
	Alcohol abuse	1 (3%)	7 (10%)	0.43
	Ischemic heart disease	8 (28%)	11 (16%)	0.27
	Hypertension	16 (55%)	31 (46%)	0.5
	Diabetes mellitus	4 (14%)	7 (10%)	0.73
	Renal function:			
	Creatinine [mg/dl]	0.86 ± 0.16	0.88 ± 0.33	0.63
	GFR ml/min [n]:			
	>90	8 (28%)	24 (36%)	0.49
	90–60	18 (62%)	35 (52%)	0.5
	60–30	2 (7%)	8 (12%)	0.72
	30–15	1 (3%)	0	0.3
Preoperative medication [n]				
	Insulin	1 (3%)	0	0.3
	Oral antidiabetics	4 (14%)	5 (8%)	0.45
	Beta-blocker	14 (48%)	18 (27%)	0.06
	Calciumantagonist	6 (21%)	10 (15%)	0.55
	ACE inhibitors	8 (28%)	12 (18%)	0.29
	ARBs blockers	1 (3%)	14 (21%)	**0.03**
	Aspirin	7 (24%)	20 (30%)	0.63
	Clopidogrel	0	1 (1,5%)	1.0
	Diuretics	3 (11%)	10 (15%)	0.75
	Antiarrhythmics	0	2 (3%)	1.0
	Bronchodilators	2 (7%)	9 (13%)	0.5
	Cortison	2 (7%)	4 (6%)	1.0
Indication for surgery [n]				
	Malignancy	19 (66%)	39 (58%)	0.65
	Metastasis	8 (28%)	15 (22%)	0.6
	Others	2 (6%)	13 (20%)	0.2
Types of surgery [n]				
	Thoracoscopy	4 (14%)	28 (42%)	**0.009**
	Thoracotomy	25 (86%)	39 (58%)	**0.009**
	Wedge resection	11 (38%)	21 (31%)	0.64
	Segmentectomy	1 (3%)	10 (25%)	0.16
	Lobectomy	15 (52%)	33 (49%)	1.0
	Pneumonectomy	2 (7%)	3 (5%)	0.64
Respiratory function [%]				
	FEV_1_	68 (54–86)	80 (66–98)	**0.008**
	VC	85 (72–104)	99 (87–111)	**0.013**
	PEF	69 (52–82)	77 (61–96)	**0.05**
	Tiffeneau-Index	64 (52–72)	64 (55–77)	0.13

Data are presented as number of patients and percentage within the group, median and interquartile range or mean and standard deviation. ASA = American Society of Anesthesiologists; Crea. = creatinine; GFR = glomerular filtration rate; ACE inhibitors = Angiotensin-converting-enzyme inhibitors; ARBs = Angiotensin-receptor-II blockers; FEV_1_ = forced expiratory volume in 1 second; VC = vital capacity; PEF = peak expiratory flow.

### Perioperative characteristics

The only perioperative parameter that differed significantly between patients with and without postoperative complications was length of hospital stay (12 (interquartile range: 11–17) vs. 9 (interquartile range: 7–11) days, *P* = 0.001). Considering the intraoperative hemodynamic parameters and therapy there was no difference between the complication and non-complication group (Table 3).

**Table 3:** Perioperative characteristics.

	Complications	No complications	*P* value
	(n = 29)	(n = 67)	
Anaesthesia [min]	280 (220–360)	245 (185–300)	0.06
Surgery [min]	183 (118–269)	163 (100–202)	0.1
Norepinephrine [μg/kg/min]	0.03 (0.02–0.04)	0.02 (0.01–0.04)	0.2
Ephedrine [n]	9 (31%)	24 (36%)	0.8
Crystalloids [ml/kg/h]	5.9 (4.2–7.7)	6.0 (4.4–7.4)	0.65
Total crystalloids [ml]	1850 (1325–3000)	2000 (1100–2100)	0.59
Urine output [ml/kg/h]	0.97 (0.75–1.6)	0.97 (0.74–1.5)	0.98
Total urine output [ml]	350 (250–510)	360 (260–600)	0.8
Total blood loss [ml]	200 (100–300)	300 (100–500)	0.2
Fluid balance [ml]	1350 (900–2380)	1300 (900–1640)	0.6
Cardiac Index [l/min/m^2^]	2.5 (2.2–3.1)	2.5 (2.0–3.2)	0.8
Mean Arterial Pressure [mmHg]	79 (75–86)	79 (75–86)	0.7
Length of hospital stay [days]	12 (11–17)	9 (7–11)	**0.001**

Data are presented as number of patients and percentage or median and interquartile range.

### Cytokine analysis

**Fig 2:**
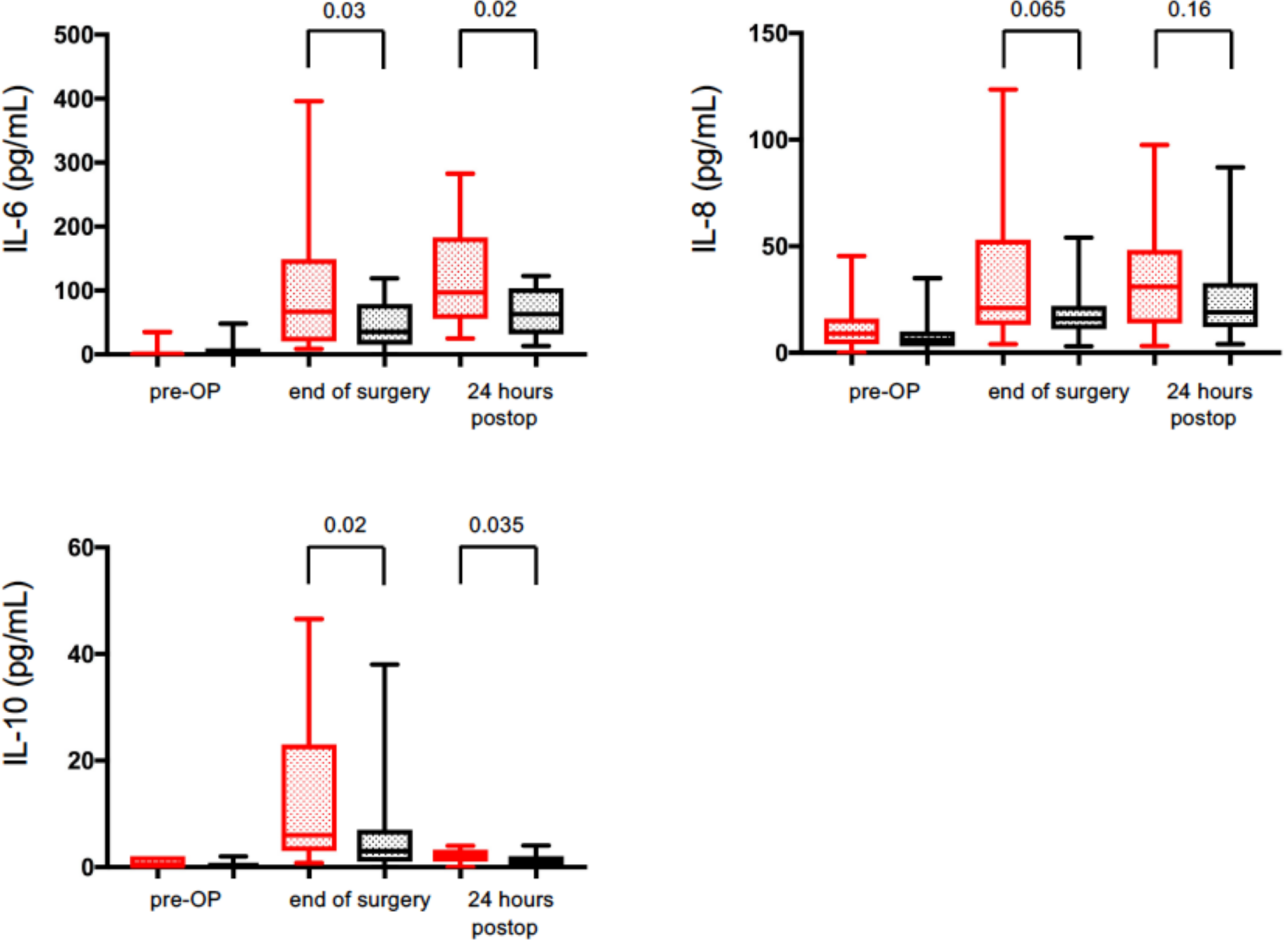
Inflammatory cytokine analysis of IL-6, IL-8 and IL-10 pre-operatively (pre-OP), at wound closure (end of surgery) and 24 hours after the end of surgery (24 hours postop). Red box plots represent the data of the complication group, black box plots represent data of the complication-free group. Data are median and interquartile range. Exact p-values are given.

Initially six different cytokines were analyzed (TNF-α, IL-1β, IL-12p70, IL-10, IL-8 and IL-6) at three different time points: before surgery, at the time of wound closure and 24 hours after surgery. Plasma concentrations of TNFα, IL-1β and IL12p70 at all three time points did not show any increase in the complication or non-complication group (data not shown). Plasma concentrations of IL-10, IL-8 and IL-6 before surgery did not differ between groups. For IL-10 the complication group showed significantly higher plasma concentration at the end of surgery and on the first postoperative day compared to the non-complication group (5.7 (interquartile range: 2.9–23) vs. 2.8 (interquartile range: 0.7–7.2) pg/ml; 2 (interquartile range: 0.7–3.3) vs. 1 (interquartile range: 0–2) pg/ml; *P* = 0.02, *P* = 0.035 respectively) (Fig 2). Patients in the complication group showed a trend to have higher plasma levels of IL-8 at the end of surgery and on the first postoperative day compared to the non-complication group (21 (interquartile range: 13–53) vs. 16 (interquartile range: 11–22) pg/ml and 31 (interquartile range: 13–49) vs. 19 (interquartile range: 12–33) pg/ml; *P* = 0.065, *P* = 0.16 respectively) (Fig 2). IL-6 plasma concentrations at the end of surgery and on the first postoperative day differed significantly. Patients in the complication group had a significantly increased IL-6 plasma concentration at the end of surgery as well as on the first postoperative day compared to the non-complication group (67 (interquartile range: 20–149) vs. 35 (interquartile range: 15–79) pg/ml and 97 (interquartile range: 56–183) vs. 63 (interquartile range: 31–103) pg/ml; *P* = 0.03, *P* = 0.02) (Fig 2).

**Fig 3:**
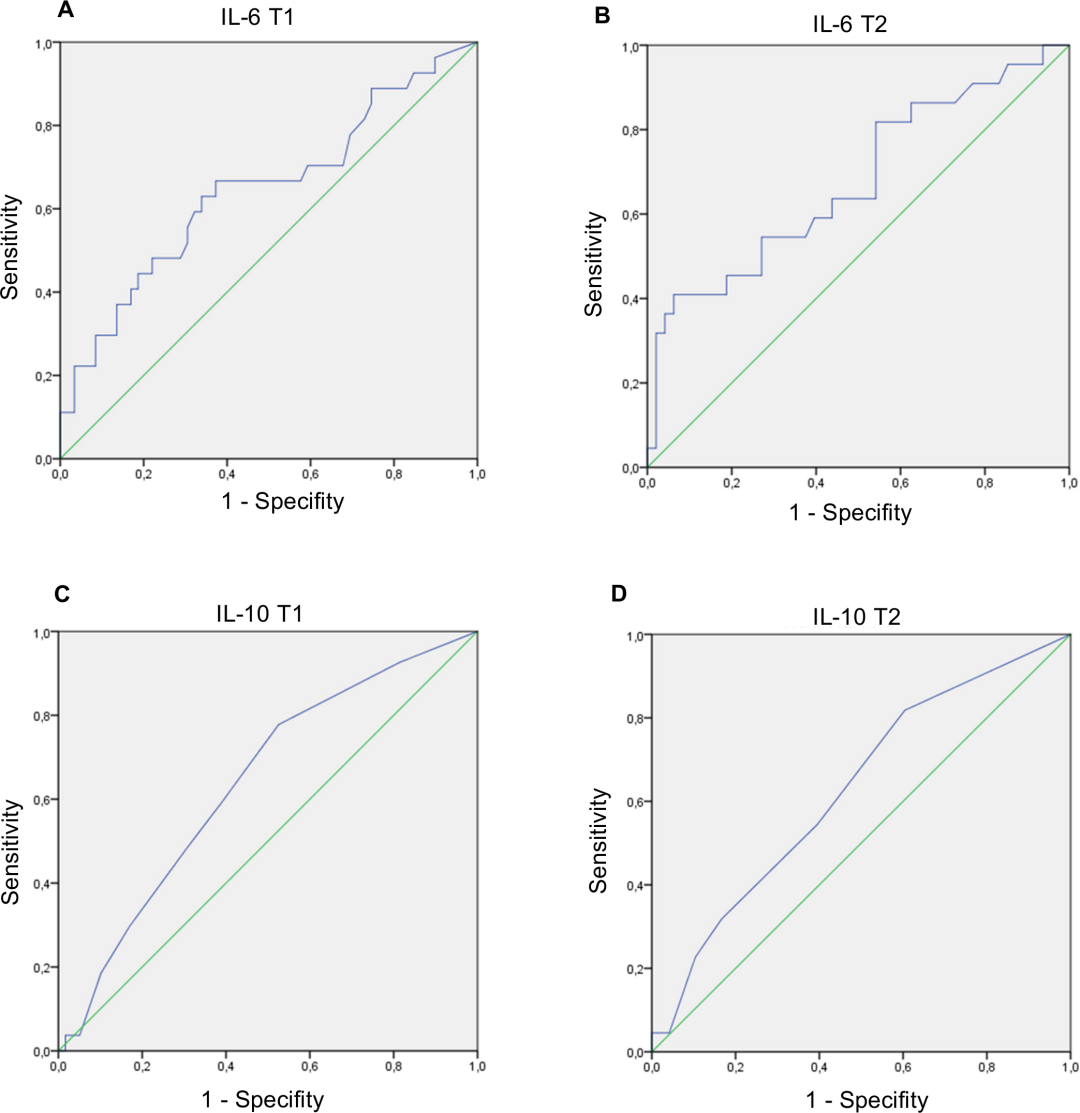
Receiver operating characteristic curves for the development of postoperative complications for inflammatory biomarkers. T1 = at wound closure; T2 = first postoperative day; (A.) area under the curve = 0.65; 95% confidence interval: 0.51–0.78; (B.) area under the curve = 0.68; 95% confidence interval: 0.54–0.82; (C.) area under the curve = 0.64; 95% confidence interval: 0.52–0.76; (D) area under the curve = 0.63; 95% confidence interval: 0.49–0.77.

To assess the quality of plasma concentrations of IL-6 at the end of surgery and on the first postoperative day to distinguish between patients with and without postoperative complications we analyzed the area under the ROC curve. Considering the IL-6 plasma concentrations at the end of surgery and on the first postoperative day the area under the ROC curve were 0.65 (95% Confidence interval (CI): 0.51–0.78) and 0.68 (95% CI: 0.54–0.82) (Fig 3). For IL-10 plasma concentrations at the end of surgery and on the first postoperative day to predict postoperative complications the area under the ROC curve were 0.64 (95% CI: 0.52–0.76) and 0.63 (95% CI: 0.49–0.77) (Fig 3). The Youden-Index for IL-6 plasma concentrations of 42.4 pg/ml at wound closure and 126.6 pg/ml on the first postoperative day to identify patients at risk of postoperative complications were 0.29 and 0.35.

As patient and baseline characteristics differ significantly between the complication and non-complication group a multivariate regression analysis was conducted to adjust the prediction of postoperative complications according to existing covariates. General postoperative complications represent the dependent variable, whereas the independent significant variables of Table 2 (FEV1, PEF, VC; nicotine, ARBs, surgical approach) are integrated as covariates. Furthermore, the numeric values for IL-10,IL-6 and IL-8 before surgery (T0), at wound closure (T1) and 24 hours after surgery (T2) are also included as independent variables in this multivariate analysis. In this analysis only the surgical approach is significant. Patients for thoracoscopic surgery had significantly less postoperative complications than patients undergoing a thoracotomy. However, the odds ratio is quite low in this case. Due to the small sample size and high interindividual variability there were no significant results for the numeric values (absolute levels) of IL-6, IL-8 and IL-10 (S2 Table).

The numeric values (absolute levels) for each cytokine show high variability between the patients which is in accordance with the results in other studies on postoperative cytokine levels. [7–9,11] Due to the high interindividual variability and small sample size we aggregated the results for each cytokine at the time of wound closure and 24 hours after surgery into its quartiles (S3 Table). We sampled all patients who had cytokine levels below the first and above the third quartile. For IL-6 and IL-10 there were no significant results whereas the surgical approach (thoracoscopy vs. thoracotomy) was significant (S4 and S5 Tables). Although the surgical approach still is significant with an Odds ratio of 0.082 favoring thoracoscopy , patients with an IL-8 level above the third quartile (75% percentile) 24 hours after surgery show a significant probability increase (P = 0.024) for postoperative complications with an Odds ratio of 7.9 (Table 4).

**Table 4:** Multivariate regression analysis for IL-8.

	Regression coefficient	P Value	Odds Ratio Exp (B)	95% Confidence interval	
FEV1	0,027	0,283	1,027	0,978	1,078
PEF	0,018	0,342	1,018	0,981	1,056
VC	-0,02	0,414	0,981	0,936	1,028
Nicotine	0,662	0,407	1,939	0,405	9,296
ARBs	-2,826	0,035	0,059	0,004	0,822
Surgical approach	-2,498	**0,009**	**0,082**	0,013	0,54
IL-8 T1 < = 1. Quartile	-1,672	0,087	0,188	0,028	1,277
IL-8 T1 > = 3. Quartile	-0,67	0,465	0,512	0,085	3,088
IL-8 T2 < = 1. Quartile	0,411	0,633	1,509	0,279	8,158
IL-8 T2 > = 3. Quartile	2,073	**0,024**	**7,948**	1,314	48,069

FEV_1_ = forced expiratory volume in 1 second; PEF = peak expiratory flow; VC = vital capacity; IL-8 = interleukin 8; T1 = at the end of surgery at wound closure; T2 = 24 hours after surgery; ARBs = Angiotensin-receptor-II blockers; surgical approach = thoracoscopy vs. thoracotomy.

In addition to the above mentioned multivariate regression analysis on each specific cytokine, we combined the levels of the two pro-inflammatory cytokines (IL-6 and IL-8) and focused on patients who had cytokine levels above the 3^rd^ quartile (75% percentile) for IL-6 and IL-8 at the end of surgery (S6 Table) and 24 hours after surgery (Table 5). Again a multivariate regression analysis was performed. General postoperative complications represent the dependent variable, whereas the independent significant variables of Table 2 (FEV1, PEF, VC; nicotine, ARBs, surgical approach) are integrated as covariates. Besides the surgical approach that showed a significant influence favoring thoracoscopy, patients who had IL-6 and IL-8 levels above the 3^rd^ quartile (75% percentile) 24 hours after surgery showed a significantly increased probability to develop postoperative complications with an Odds ratio of 56.4 (Table 5). We performed the same multivariate regression analysis by focusing on patients who had IL-6, IL-8 and IL-10 levels above the 3^rd^ quartile. Actually there were three patients who fulfilled this condition and all three patients suffered from postoperative complications. However, the amount of patients is too low to conduct a reliable multivariate regression analysis on this combined outcome (IL-6, IL-8 and IL-10).

**Table 5:** Multivariate regression analysis for patients with IL-8 and IL-6 levels above the 3^rd^ quartile 24 hours after surgery.

	Regression coefficient	P Value	Odds Ratio Exp (B)	95% Confidence interval	
FEV1	0,035	0,151	1,036	0,987	1,087
PEF	0,009	0,643	1,009	0,973	1,046
VC	-0,032	0,193	0,968	0,923	1,016
Nicotine	0,776	0,296	2,173	0,506	9,321
ARBs	-2,798	0,077	0,061	0,003	1,352
Surgical approach	-2,972	**0,018**	**0,051**	0,004	0,598
IL-6 T2 & IL-8 T2 > = 3. Quartile	4,033	**0,013**	**56,436**	2,32	1372,701

FEV_1_ = forced expiratory volume in 1 second; PEF = peak expiratory flow; VC = vital capacity; IL-6 = interleukin 6; IL-8 = interleukin 8; T2 = 24 hours after surgery; ARBs = Angiotensin-receptor-II blockers; surgical approach = thoracoscopy vs. thoracotomy.

## Discussion

In this prospective, clinical study we demonstrated that increased IL-6, IL-8 and IL-10 levels at an early stage after lung surgery might be of essential prognostic value to identify patients at risk for postoperative complications.

The main results of this study can be summarized as follows. First, the multivariate regression analysis showed that patients whose IL-8 levels were above the 3^rd^ quartile 24 hours after surgery and especially those with a combined increase of IL-8 and IL-6 above the 3^rd^ quartile bear a significantly increased risk for postoperative complications. Second, in a simplified model not taking into account other existing covariates, plasma concentrations of IL-10 and IL-6 are significantly increased at the time of wound closure and on the first postoperative day in patients undergoing non-cardiac thoracic surgery. Third, the incidence of postoperative complications in this study was 34, whereas the total amount of patients with postoperative complications was 29. Fourth, patients’ demographic and surgical characteristics were assessed to verify further risk factors for postoperative complications such as nicotine dependency, ARBs, surgical approach and preoperative respiratory function. Finally, patients with complications had an increased length of hospital stay despite comparable intraoperative characteristics according to the hemodynamic management including fluid therapy and time for surgery.

Our results regarding plasma concentrations of IL-10, IL-8 and IL-6 in patients undergoing lung resection resemble the concentrations and standard deviations highlighted in other studies on lung surgery.[8,9,13,17] In response to one-lung ventilation with ischemia/reperfusion injury of the lungs, parenchyma resection and surgical approach pro- and anti-inflammatory cytokines (IL-6, IL-8; IL-10) are released.[10] There are some studies analyzing the inflammatory response after thoracic surgery, however they did not focus on postoperative complications just on the postoperative systemic inflammatory response syndrome.[7–9] In contrast to our results, the authors were not able to show significantly increased plasma IL-6 levels after surgery in patients with postoperative complications.[13] The surgical approach (thoracotomy vs. thoracoscopy) was defined as a risk factor in several studies. Patients undergoing lung surgery via thoracotomy had a higher rate of postoperative complications and increased plasma concentrations of IL-6, IL-8 and IL-10.[18,19] In accordance with these results patients in the complication group with higher plasma concentrations of IL-6, IL-8 and IL-10 underwent thoracotomy more often (86% versus 58%). We measured IL-6 and IL-10 plasma concentrations at the time of wound closure and on the first postoperative day as most complications occur quite early on the second or even first postoperative day.[4,16,20,21] To evaluate the measurement of plasma concentrations of IL-6 and IL-10 at the end of surgery and on the first postoperative day to discriminate between patients who will develop postoperative complications and those who will not, ROC curve analysis was performed and the Youden Index was calculated for IL-6 as the most promising inflammatory biomarker. The area under the curve revealed moderate prediction capacity of IL-6 and IL-10 for postoperative complications. The Youden index calculated for IL-6 plasma concentrations to distinguish between patients with and without postoperative complications within 30 days after surgery also showed moderate prediction quality of calculated thresholds at the time of wound closure and 24 hours after surgery. The main disadvantage of this statistic model is the fact that existing covariates are not taken into consideration for risk stratification. Therefore, multivariate regression analysis was conducted revealing a significant probability increase for postoperative complications if IL-8 or IL-8 in combination with IL-6 is increased above the 3^rd^ quartile of this cohort on the first postoperative day.

The early inflammatory response after surgery may be the origin of general postoperative organ dysfunction.(11) In contrast to the initial study focusing on goal-directed therapy, group division into two groups was based on general postoperative complications as a composite outcome due to the fact that postoperative complications are linked to each other. Postoperative occurrence of early acute kidney injury is associated with cardiopulmonary complications, for example.[16] The incidences of postoperative complications in this study, especially pulmonary complications, are in agreement with previous studies.[1,4,16] However, due to the small sample size and high variability in cytokine response focusing on only one type of complication would not be valuable. As a consequence, with regards to the increase of postoperative inflammatory cytokines we focused on all complications.

Several studies have been conducted analyzing predictors of mortality and morbidity for patients undergoing lung surgery, however inflammatory biomarkers were not included.[4,6,21] In this study comparison of patients’ and surgical characteristics between the two groups resemble the results of these studies.

In this prospective, clinical study intraoperative hemodynamic therapy and parameters did not differ between patients with and without postoperative complications. In formerly published data on lung surgery patients, more than 2500 ml of intraoperative fluids was reported to be a risk factor for postoperative complications.[22] In our complication group, patients experienced a reasonable fluid administration with a mean of less than 2500 ml. Both groups, complication and non-complication group, showed sufficient cardiac output and mean arterial pressure. Intraoperative ventilation management was lung protective and conducted according to the study protocol. With regards to the intraoperative management and parameters it was not obvious at the end of surgery which patient bears an increased risk to develop postoperative complications from clinical data only.

There are several limitations of this prospective clinical study. Although intraoperative respiratory and hemodynamic parameters did not differ between the two groups all cytokines show high standard deviations representing the variability of inflammatory response in patients undergoing lung surgery. Previous studies on cytokine expression in lung surgical patients showed similar results with regards to plasma concentrations and high standard deviations.[7,11–13] Several studies have been conducted on preoperative and intraoperative risk factors but our primary aim was to add an early detection biomarker to already pre-described risk factors for lung surgical patients. Compared to other cytokine studies on lung surgery patients we did not measure cytokine levels in the bronchial alveolar fluids which might have confirmed our results on systemic acting cytokines, however focusing on general postoperative complications the detection of a localized inflammatory response might not be reasonable. We did not measure cytokine plasma concentrations beyond the first postoperative day as the expression of cytokines show a maximum at that time.[7–9,11] For the multivariate regression analysis due to the high interindividual variability and small sample size we aggregated the results for each cytokine at the time of wound closure and 24 hours after surgery into its quartiles. We sampled all patients who had cytokine levels below the first and above the third quartile. The cohort of this study used to determine the different quartile ranges is small so that a bigger amount of patients is needed to determine a reliable threshold for daily clinical practice.

Identifying patients at risk for postoperative complications as early as possible might help to prevent these by taking those patients into a preventive program with an extended respiratory therapy or increased attention on goal-directed postoperative hemodynamic management, for example. As hospital resources are limited, the use of preventive measures which are time consuming and expensive needs to be well justified.

## Conclusions

The multivariate regression analysis of lung surgery patients with increased IL-8 and IL-6 levels above the 3^rd^ quartile on the first postoperative day revealed a significant probability increase for postoperative complications. Apart from that, patients with postoperative complications showed a significantly increased IL-6 and IL-10 plasma concentration already at the time of wound closure compared to those without complications. Evaluation of IL-6 as a predictive biomarker showed moderate quality as a single measurement at the time of wound closure or first postoperative day. A reasonable approach to quantify patients’ individual risk for postoperative complications might be the combination of clinical pre-/intraoperative characteristics and the early detection of increased inflammatory biomarkers.

## Supporting information

S1 File Study protocol approved by the local Ethics Committee. (DOCX)

S2 File Consort checklist. (DOC)

S1 Table Postoperative complications. GDT = Goal-directed therapy. Number of patients and percentage within each group.
(DOCX)

S2 Table Multivariate regression analysis of absolute interleukin levels. FEV_1_ = forced expiratory volume in 1 second; PEF = peak expiratory flow; VC = vital capacity; IL-10 = interleukin 10; IL-8 = interleukin 8; IL-6 = interleukin 6; T0 = Before surgery; T1 = at the end of surgery at wound closure; T2 = 24 hours after surgery; ARBs = Angiotensin-receptor-II blockers; surgical approach (thoracoscopy versus thoracotomy).
(DOCX)

S3 Table Aggregation of interleukin levels into quartiles. IL-10 = interleukin 10; IL-8 = interleukin 8; IL-6 = interleukin 6; T0 = Before surgery; T1 = at the end of surgery at wound closure; T2 = 24 hours after surgery.
(DOCX)

S4 Table Multivariate regression analysis for IL-6. FEV_1_ = forced expiratory volume in 1 second; PEF = peak expiratory flow; VC = vital capacity; IL-6 = interleukin 6; T0 = Before surgery; T1 = at the end of surgery at wound closure; T2 = 24 hours after surgery; ARBs = Angiotensin-receptor-II blockers; surgical approach (thoracoscopy versus thoracotomy).
(DOCX)

S5 Table Multivariate regression analysis for IL-10. FEV_1_ = forced expiratory volume in 1 second; PEF = peak expiratory flow; VC = vital capacity; IL-10 = interleukin 10; T0 = Before surgery; T1 = at the end of surgery at wound closure; T2 = 24 hours after surgery; ARBs = Angiotensin-receptor-II blockers; surgical approach (thoracoscopy versus thoracotomy).
(DOCX)

S6 Table Multivariate regression analysis for patients with IL-8 and IL-6 levels above the 3^rd^ quartile at the end of surgery. FEV_1_ = forced expiratory volume in 1 second; PEF = peak expiratory flow; VC = vital capacity; IL-6 = interleukin 6; IL-8 = interleukin 8; T1 = at the end of surgery at wound closure; ARBs = Angiotensin-receptor-II blockers; surgical approach (thoracoscopy versus thoracotomy.
(DOCX)
