# Supplementary material for: Perioperative cytokine profile during lung surgery predicts patients at risk for postoperative complications—A prospective, clinical study
Source: PLoS One. 2018 Jul 3;13(7):e0199807. doi: 10.1371/journal.pone.0199807 (PMC6029786; doi:10.1371/journal.pone.0199807)
Supplement: S1 File — (DOCX) [file pone.0199807.s001.docx]

**Study protocol for a randomized-controlled trial**

**(Version 2.0)**

Advanced hemodynamic monitoring with esophageal Doppler monitoring for patients undergoing lung resection surgery to reduce postoperative pulmonary complications

**Investigators:**

Dr. Kai Kaufmann, Prof. Torsten Loop, Prof. Dr. Ulrich Göbel

Department of Anesthesiology and Critical Care

Medical Center - University of Freiburg, Faculty of Medicine

Hugstetter Strasse 55, 79106 Freiburg im Breisgau, Germany

Tel.: +49-761-270-23060

Fax: +49-761-270-23960

**Research question**

This randomized controlled trial focuses on patients undergoing surgery for lung parenchymal resection. After randomization the intraoperative hemodynamic changes are measured by esophageal Doppler monitoring. Patients are divided into two groups, intervention group versus control group. In both groups esophageal Doppler monitoring is installed to measure hemodynamic parameters according to the aortic blood flow.

In the control group the anesthesiologist in charge has no insights into the esophageal Doppler monitoring data. In the intervention group the anesthesiologist is aware of the esophageal Doppler monitoring data and hemodynamic management is guided according to the additional information.

Primary endpoint is the incidence of postoperative pulmonary complications. Secondary endpoints include postoperative acute kidney injury and cytokine expression measured in patients’ blood samples.

**Background**

The intraoperative hemodynamic management is one of the main challenges in anesthesiology. By using intravenous fluids and inotropic agents a sufficient cardiac output and hence an adequate organ perfusion should be guaranteed. One of the major intraoperative goals is the arterial blood pressure as a static variable, whereas dynamic parameters are usually not applicable. The ideal intraoperative hemodynamic management in correspondence to patients’ and surgical characteristics is crucial for patients’ outcome.^1^ Hyper- and hypovolemia can have a detrimental effect on patients’ survival after surgery.^2,3^ Consequences of intraoperative hypovolemia are hemodynamic instability, insufficient organ perfusion and consecutive organ dysfunction.^2,3^ The postoperative incidence of prerenal acute kidney injury among patients after non-cardiac thoracic surgery due to hypovolemia is 5.9 %.^4^ On the other hand intraoperative hypervolemia leads to a significant deterioration of patients’ outcome in non-cardiac thoracic surgery.^5-8^

The main postoperative complications in non-cardiac thoracic surgery are of pulmonary origin: pneumonia, atelectasis, respiratory insufficiency, bronchopleural fistula, pleural effusion, acute respiratory distress syndrome (ARDS)

Unspecific fluid administration as part of the intraoperative hemodynamic management needs to be verified.^9^ Goal-directed fluid and catecholamine therapy according to dynamic parameters, i.e. aortic blood flow, constitutes the basis of modern intraoperative, hemodynamic management. According to this strategy individualized thresholds for static and dynamic parameters were defined and used as surrogates for sufficient organ perfusion. Static hemodynamic parameters include: Central venous pressure (CVP); Mean arterial pressure (MAP); pulmonary-capillary wedge pressure (PCWP). Dynamic parameters include: Pulse pressure (PP) and stroke volume (SV). To evaluate and control hemodynamic changes individually, static parameters appear insufficient.^10,11^

One option to determine stroke volume is the esophageal Doppler monitoring (EDM) measuring the aortic blood flow using the Doppler effect. The CardioQ^TM^ system consists of a Doppler probe which is inserted into the esophagus, blood flow velocity in the aorta and cardiac output are measured continuously.^12,13^ Various studies showed the superiority of the esophageal Doppler monitoring as a continuous hemodynamic measurement device in contrast to the intermittent options given by the pulmonary artery catheter.^14^ The comparison of the non-invasive esophageal Doppler monitoring in non-cardiac surgery patients with other established methods (pulse contour continuous cardiac output, PICCO; pulmonary artery catheter, PAC) showed a decreased invasiveness with less complications.^14^ Apart from that, non-calibrated methods (Lidco-Rapid®; Flowtrac/Vigileo®) appear insufficient.^15^ Applying the esophageal Doppler monitoring in non-cardiac thoracic patients revealed a sensitive detection of a stroke volume reduction before changes in heart rate and arterial blood pressure were detected. ^14^ In non-cardiac surgery patients the use of an EDM based GDT reduced patients’ hospital stay, mortality and morbidity rate. ^16-19^

The National Institute for Health and Care Excellence (NICE) in England include the CardioQ^TM^ system into their guidelines to improve the intraoperative hemodynamic management of critically ill patients.^20^ The German Society of Anesthesiology and Critical Care published S3-guidelines for cardiac surgery patients on hemodynamic monitoring.^21^These S3-guidelines cannot be transferred to other high risk patients. Guidelines on hemodynamic management for other high risk groups are still pending.

The use of EDM for intraoperative hemodynamic management in non-cardiac thoracic patients to reduce the rate of postoperative complications has not yet been investigated.

**Hypothesis**

The use of EDM for patients undergoing non-cardiac thoracic surgery leads to a better outcome compared to those with standard monitoring.

**Methods**

**(1) Patients**

Written informed consent was obtained for participation in the research discussed in this manuscript from every patient enrolled in this study. Patients older than 18 years with lung parenchymal resection in general anesthesia under one-lung ventilation are going to be enrolled. Patients are randomized into an intervention and control group. In both groups EDM is installed. In the control group EDM data are just recorded but not visible to the anesthesiologist in charge. In the intervention group the anesthesiologist in charge uses the EDM data to optimize patients’ hemodynamic status. Patients of the control group receive standard hemodynamic monitoring (ECG, arterial blood pressure, peripheral oxygen saturation, urine output, central venous pressure, blood gas analysis), whereas the intervention group has standard monitoring plus EDM. Routinely, all patients were admitted to our hospital the day before surgery to receive standard preoperative evaluation (blood draw, spirometry).

Both groups underwent the same intraoperative anesthesiological management consisting of a combination of epidural and general anesthesia. The type of intraoperative medication for anesthesia and hemodynamic management was the same in both groups.

Randomization


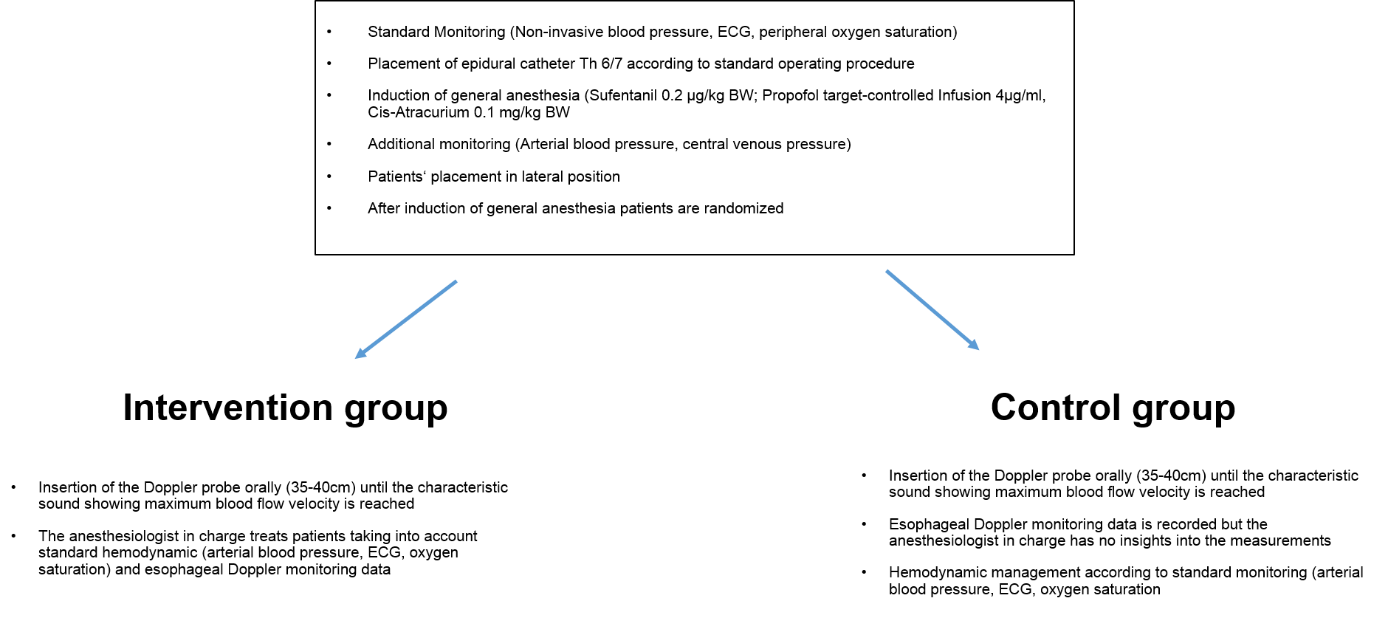


The postoperative treatment on ICU is identical in both groups, not study protocolized and was done in accordance with standard clinical care. The following postoperative parameters were recorded: vigilance, heart rate, arterial blood pressure, peripheral oxygen saturation, catecholamines, pain level (NRS), urine output, amount of pleural effusion. Further parameters are mentioned under the study protocol section.

**(2) Esophageal Doppler monitoring**

The CardioQ^TM^ system (Deltex Medical, Chichester, West Sussex, UK) consists of a Doppler probe inserted into the esophagus and a monitor including a computer to calculate and display the hemodynamic parameters. This is a medical device with a CE-labeling, furthermore no additional intended purpose of this product will be determined in this study. The diameter of the Doppler probe is 6 mm and is inserted orally (35-40cm). The correct placement of the Doppler probe can be verified by a specific signal that reaches a maximum where aorta and esophagus are located parallel to each other. Blood flow velocity is calculated by the Doppler effect using the frequency shift from the emitted and receiving ultrasonic waves. The aortic diameter is extrapolated from a nomogram including patients’ age, height and weight. The multiplication of the area under the velocity-time-curve with the aortic diameter results in patient’s stroke volume (SV). Heart rate, SV and body mass are used to calculate patients’ cardiac index (l/min/m²). In both groups the esophageal Doppler probe is placed after induction of general anesthesia.

**(3) Data acquisition**

The following paragraph highlights the collection of data and distinguishes between data that are routinely acquired and those that are documented due to study protocol.

Preoperative data:

1. Demographics (routine):

Height, weight, age, gender, medication, diseases, ASA classification

1. Spirometry (routine):

Forced-expiratory-volume in 1 sec. (FEV1), Peak Expiratory Flow (PEF), Vital capacity (VC), Inspiratory reserve volume (IRV), Expiratory reserve volume (ERV)

1. Blood gas analysis (routine):

pH, oxygen partial pressure, CO_2_ partial pressure, base excess, standard bicarbonate, lactat

1. Renal parameters (routine):

Creatinine, Urea

Intraoperative data recorded:

1. Average and maximum dose of norepinephrine (µg/kg/min)
2. Average and maximum dose of epinephrine (µg/kg/min)
3. Blood loss (ml)
4. Blood gas analysis to evaluate gas exchange; in addition this routine blood sample is used to measure the cytokine expression before surgical incision after general anesthesia induction and at the end of surgery at the time of wound closure. (The additional measurement of cytokines **does not** lead to an increased blood drawing)
5. Amount of intravenous fluids cristalloids/kolloids (ml)
6. Urine output (ml/kg/h)
7. EDM data acquisition according to study protocol:
   1. Stroke volume (SV)
   2. Cardiac output (CO)
   3. Cardiac index (CI)
   4. Stroke volume variation (SVV)
   5. Peak velocity (PV)
   6. Flow time (FT)
   7. Systemic vascular resistance (SVR)

Intraoperative EDM Data are only visible to anesthesiologists treating patients of the intervention group. Anesthesiologists treating patients of the control group have no insights into intraoperative EDM data.

Data collection 24 and 72 hours after surgery by a research personnel unaware of group assignment

1. Appraisal of chest X-Ray by radiologist unaware of group assignment (routine): infiltrates, atelectasis, dystelectasis, pleural effusion
2. Amount of thoracic drainage (routine)
3. Bronchopleural fistula (routine)
4. Pain level (NRS) (routine)
5. Spirometry (study protocol): Forced-expiratory-volume in 1 sec. (FEV1), Peak Expiratory Flow (PEF), Vital capacity (VC), Inspiratory reserve volume (IRV), Expiratory reserve volume (ERV)
6. Blood gas analysis (routine):

pH, oxygen partial pressure, CO_2_ partial pressure, base excess, standard bicarbonate, lactat

1. Renal parameters (routine):

Creatinine, Urea

1. Acute kidney injury according to AKI criteria^4^
2. Catecholamines (routine)
3. Peripheral oxygen saturation SpO_2_ (routine):
   1. SpO_2_ < 90% at room air
   2. SpO_2_ < 88% at room air
4. Blood gas analysis to evaluate gas exchange; in addition this routine blood sample is used to measure the cytokine expression

**Study protocol**

**Day before surgery**

Obtaining informed consent for study assignment of the patient and of the consultant anesthesiologist in charge taking care of the patient intraoperatively, spirometry (Forced-expiratory-volume in 1 sec. (FEV1), Peak Expiratory Flow (PEF), Vital capacity (VC), Inspiratory reserve volume (IRV), Expiratory reserve volume (ERV)); Blood gas analysis (routine): pH, oxygen partial pressure, CO_2_ partial pressure, base excess, standard bicarbonate, lactat; blood draw (routine): renal parameters

The study protocol of the preoperative day does not include any differences between the intervention and control group. All the examinations which are done on this day belong to the routine diagnostics.

**Day of surgery**

Both groups receive the same kind of intraoperative medication according to current clinical practice. However, patients of the intervention group were managed according to EDM data.


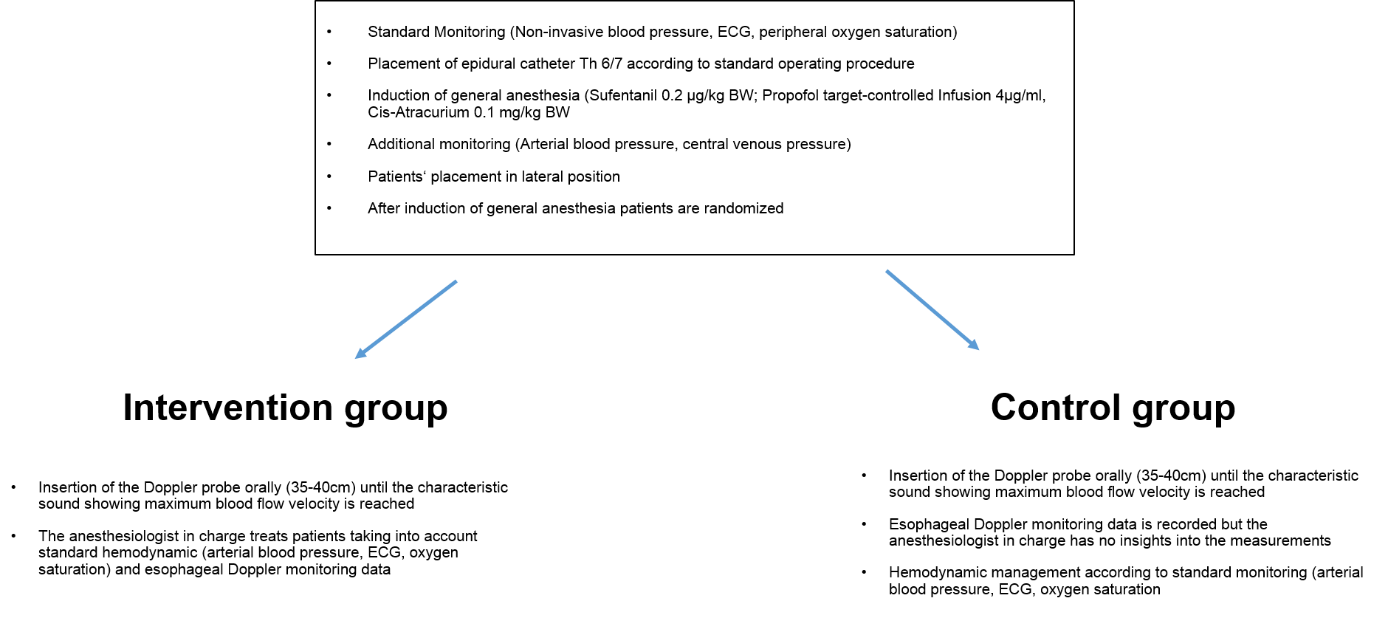


**Intraoperative** data recorded:

1. Average and maximum dose of norepinephrine (µg/kg/min)
2. Average and maximum dose of epinephrine (µg/kg/min)
3. Blood loss (ml)
4. Blood gas analysis to evaluate gas exchange; in addition this routine blood sample is used to measure the cytokine expression before surgical incision after general anesthesia induction and at the end of surgery at the time of wound closure. (The additional measurement of cytokines **does not** lead to an increased blood drawing; only 50µl are needed for cytokine analysis)
5. Amount of intravenous fluids cristalloids/kolloids (ml)
6. Urine output (ml/kg/h)
7. EDM data acquisition according to study protocol:
   1. Stroke volume (SV)
   2. Cardiac output (CO)
   3. Cardiac index (CI)
   4. Stroke volume variation (SVV)
   5. Peak velocity (PV)
   6. Flow time (FT)
   7. Systemic vascular resistance (SVR)

Intraoperative EDM Data are only visible to anesthesiologists treating patients of the intervention group. Anesthesiologists treating patients of the control group have no insights into intraoperative EDM data.

**24 hours** after surgery: The postoperative treatment on ICU was identical in both groups in correspondence with our standard operating procedures, staff on the ward was unaware of group assignment.

1. Appraisal of chest X-Ray by radiologist unaware of group assignment (routine): infiltrates, atelectasis, dystelectasis, pleural effusion
2. Amount of thoracic drainage (routine)
3. Bronchopleural fistula (routine)
4. Pain level (NRS) (routine)
5. Spirometry (study protocol): Forced-expiratory-volume in 1 sec. (FEV1), Peak Expiratory Flow (PEF), Vital capacity (VC), Inspiratory reserve volume (IRV), Expiratory reserve volume (ERV)
6. Blood gas analysis (routine):

pH, oxygen partial pressure, CO_2_ partial pressure, base excess, standard bicarbonate, lactat; this routine blood sample is used to measure the cytokine expression 24 hours after surgery (The additional measurement of cytokines **does not** lead to an increased blood drawing; only 50µl are needed for cytokine analysis)

1. Renal parameters (routine):

Creatinine, Urea

1. Acute kidney injury according to AKI criteria^4^
2. Catecholamines (routine)
3. Peripheral oxygen saturation SpO_2_ (routine):
   1. SpO_2_ < 90% at room air
   2. SpO_2_ < 88% at room air

**72 hours** after surgery: The postoperative treatment on ICU was identical in both groups in correspondence with our standard operating procedures, staff on the ward was unaware of group assignment.

1. Appraisal of chest X-Ray by radiologist unaware of group assignment (routine): infiltrates, atelectasis, dystelectasis, pleural effusion
2. Amount of thoracic drainage (routine)
3. Bronchopleural fistula (routine)
4. Pain level (NRS) (routine)
5. Spirometry (study protocol): Forced-expiratory-volume in 1 sec. (FEV1), Peak Expiratory Flow (PEF), Vital capacity (VC), Inspiratory reserve volume (IRV), Expiratory reserve volume (ERV)
6. Blood gas analysis (routine):

pH, oxygen partial pressure, CO_2_ partial pressure, base excess, standard bicarbonate, lactat; this routine blood sample is used to measure the cytokine expression 72 hours after surgery (The additional measurement of cytokines **does not** lead to an increased blood drawing; only 50µl are needed for cytokine analysis)

1. Renal parameters (routine):

Creatinine, Urea

1. Acute kidney injury according to AKI criteria^4^
2. Catecholamines (routine)
3. Peripheral oxygen saturation SpO_2_ (routine):
   1. SpO_2_ < 90% at room air
   2. SpO_2_ < 88% at room air

**General information**

**Study site:**

Department of Anesthesiology and Critical Care

Medical Center - University of Freiburg, Faculty of Medicine

Hugstetter Strasse 55, 79106 Freiburg im Breisgau, Germany

**Study design:**

Monocentric, prospective, randomized-controlled trial

**Number of patients:**

Statistic and biometric consulting done by Dr. Manfred Olschewski (IMBI, Institute of Medical Biometry and Statistics University of Freiburg, Freiburg, Germany) on the 2^nd^ of October 2014; total number of patients: 96; 48 patients per group. Sample size calculation was based on a reported incidence of PPCs after lung surgery of approximately 24%. We aimed to detect a reduction in the PPC rate to 3% with a power of 80% and a two-sided significance level of 5%, the calculated sample size

was 48 subjects per group.

**Inclusion criteria:**

1. Age older than 18 years
2. Formal informed consent
3. General anesthesia with endotracheal double-lumen tube intubation and one-lung ventilation
4. Lung parenchyma resection

**Exclusion criteria:**

1. Implantable cardioverter defibrillator or pacemaker
2. Age < 18 years
3. Emergency surgery
4. New York Heart Association Functional Classification 4
5. Morbid obesity (body mass index [BMI] > 50 kg m^-2^)
6. Esophageal pathologies I
7. Intraoperative blood loss of more than 1.5 liter
8. Pregnancy
9. Intraoperative use of diuretics
10. Cardiac valve pathologies
11. Intraoperative blood transfusion

**Patients’ medical risks**

Patients’ medical risks in this randomized-controlled trial are very low. The Doppler probe is soft and elastic, the probability to cause injury during probe insertion is very low. The time course of patients participating in this study does not differ from the normal standard operating procedure, time for anesthesia or surgery are not prolonged. There are no additional blood draws or chest x-rays, apart from the standard of care in our hospital. During the whole procedure consultant anesthesiologists are taking care of the patient. Patients randomized to the intervention group receive medical intention by a consultant anesthesiologist and a research personnel who is responsible for data recording during surgery so that the anesthesiologist in charge is not distracted by additional documentation.

**Literature**

1. Chau, E.H.L. & Slinger, P. Perioperative Fluid Management for Pulmonary Resection Surgery and Esophagectomy. in *Seminars in cardiothoracic and vascular anesthesia* 01089253213491014 (SAGE Publications, 2013).

2. Mythen, M. & Webb, A. Intra-operative gut mucosal hypoperfusion is associated with increased post-operative complications and cost. *Intensive care medicine* **20**, 99-104 (1994).

3. Mythen, M.G. & Webb, A.R. Perioperative plasma volume expansion reduces the incidence of gut mucosal hypoperfusion during cardiac surgery. *Archives of Surgery* **130**, 423 (1995).

4. Ishikawa, S., Griesdale, D.E. & Lohser, J. Acute kidney injury after lung resection surgery: incidence and perioperative risk factors. *Anesthesia & Analgesia* **114**, 1256-1262 (2012).

5. Fernández-Pérez, E.R., Keegan, M.T., Brown, D.R., Hubmayr, R.D. & Gajic, O. Intraoperative tidal volume as a risk factor for respiratory failure after pneumonectomy. *Anesthesiology* **105**, 14-18 (2006).

6. Marret, E.*, et al.* Risk and protective factors for major complications after pneumonectomy for lung cancer. *Interactive cardiovascular and thoracic surgery* **10**, 936-939 (2010).

7. Alam, N.*, et al.* Incidence and risk factors for lung injury after lung cancer resection. *The Annals of thoracic surgery* **84**, 1085-1091 (2007).

8. Licker, M.*, et al.* Risk factors for acute lung injury after thoracic surgery for lung cancer. *Anesthesia & Analgesia* **97**, 1558-1565 (2003).

9. Holte, K.*, et al.* Liberal or restrictive fluid administration in fast-track colonic surgery: a randomized, double-blind study†. *British journal of anaesthesia* **99**, 500-508 (2007).

10. Hansen, R.M.*, et al.* Poor correlation between pulmonary arterial wedge pressure and left ventricular end-diastolic volume after coronary artery bypass graft surgery. *Anesthesiology* **64**, 764-770 (1986).

11. Kumar, A.*, et al.* Pulmonary artery occlusion pressure and central venous pressure fail to predict ventricular filling volume, cardiac performance, or the response to volume infusion in normal subjects. *Critical care medicine* **32**, 691-699 (2004).

12. Cholley, B.P. & Singer, M. Esophageal Doppler: noninvasive cardiac output monitor. *Echocardiography* **20**, 763-769 (2003).

13. Feldheiser, A.*, et al.* Development and Feasibility Study of an Algorithm for Intraoperative Goal-Directed Haemodynamic Management in Noncardiac Surgery. *Journal of International Medical Research* **40**, 1227-1241 (2012).

14. Diaper, J.*, et al.* Transoesophageal Doppler monitoring for fluid and hemodynamic treatment during lung surgery. *Journal of clinical monitoring and computing* **22**, 367-374 (2008).

15. Wittkowski, U.*, et al.* Hämodynamisches Monitoring in der perioperativen Phase. *Der Anaesthesist* **58**, 764-786 (2009).

16. Gan, T.J.*, et al.* Goal-directed intraoperative fluid administration reduces length of hospital stay after major surgery. *Anesthesiology* **97**, 820-826 (2002).

17. Sinclair, S., James, S. & Singer, M. Intraoperative intravascular volume optimisation and length of hospital stay after repair of proximal femoral fracture: randomised controlled trial. *Bmj* **315**, 909-912 (1997).

18. Venn, R.*, et al.* Randomized controlled trial to investigate influence of the fluid challenge on duration of hospital stay and perioperative morbidity in patients with hip fractures†. *British Journal of Anaesthesia* **88**, 65-71 (2002).

19. Walsh, S., Tang, T., Bass, S. & Gaunt, M. Doppler‐guided intra‐operative fluid management during major abdominal surgery: systematic review and meta‐analysis. *International journal of clinical practice* **62**, 466-470 (2008).

20. Ghosh, S., Arthur, B. & Klein, A. NICE guidance on CardioQTM oesophageal Doppler monitoring. *Anaesthesia* **66**, 1081-1083 (2011).

21. Carl, M.*, et al.* S3 guidelines for intensive care in cardiac surgery patients: hemodynamic monitoring and cardiocirculary system. *GMS German Medical Science* **8**(2010).

**S2 Study protocol**
