# Supplementary material for: Perioperative cytokine profile during lung surgery predicts patients at risk for postoperative complications—A prospective, clinical study
Source: PLoS One. 2018 Jul 3;13(7):e0199807. doi: 10.1371/journal.pone.0199807 (PMC6029786; doi:10.1371/journal.pone.0199807)
Supplement: S1 Table — GDT = Goal-directed therapy. Number of patients and percentage within each group. (DOCX) [file pone.0199807.s003.docx]

|  | GDT |  | Control |  | *P* Value |
| --- | --- | --- | --- | --- | --- |
|  | (n=48) |  | (n=48) |  |  |
|  |  |  |  |  |  |
| Atelectasis | 3 (6) |  | 7 (14) |  | 0.32 |
|  |  |  |  |  |  |
| Pneumonia | 2 (4) |  | 4 (8) |  | 0.68 |
|  |  |  |  |  |  |
| Pleural empyema | 1 (2) |  | 1 (2) |  | 1.0 |
|  |  |  |  |  |  |
| Respiratory failure | 0 |  | 2 (4) |  | 0.5 |
|  |  |  |  |  |  |
| Pulmonary embolism | 0 |  | 2 (4) |  | 0.5 |
|  |  |  |  |  |  |
| Broncho-pleural fistula | 0 |  | 1 (2) |  | 1.0 |
|  |  |  |  |  |  |
| Renal | 2 (4) |  | 1 (2) |  | 1.0 |
|  |  |  |  |  |  |
| Cardiac | 3 (6) |  | 3 (6) |  | 1.0 |
|  |  |  |  |  |  |
| Neurological | 0 |  | 2 (4) |  | 0.5 |
|  |  |  |  |  |  |
| Total complications | 11 (23) |  | 23 (48) |  | 0.018 |
|  |  |  |  |  |  |
| Total amount of patients with complications | 11 (23) |  | 18 (38) |  | 0.13 |

S3 Postoperative complications. GDT = Goal-directed therapy. Number of patients and percentage within each group
