# Supplementary material for: Perioperative cytokine profile during lung surgery predicts patients at risk for postoperative complications—A prospective, clinical study
Source: PLoS One. 2018 Jul 3;13(7):e0199807. doi: 10.1371/journal.pone.0199807 (PMC6029786; doi:10.1371/journal.pone.0199807)
Supplement: S2 Table — FEV1 = forced expiratory volume in 1 second; PEF = peak expiratory flow; VC = vital capacity; IL-10 = interleukin 10; IL-8 = interleukin 8; IL-6 = interleukin 6; T0 = Before surgery; T1 = at the end of surgery at wound closure; T2 = 24 hours after surgery; ARBs = Angiotensin-receptor-II blockers; surgical approach (thoracoscopy versus thoracotomy). (DOCX) [file pone.0199807.s004.docx]

|  | Regression coefficient | P Value | Odds Ratio | 95% Confidence interval | |
| --- | --- | --- | --- | --- | --- |
| FEV1 | 0,039 | 0,238 | 1,04 | 0,974 | 1,111 |
| PEF | 0,017 | 0,446 | 1,017 | 0,974 | 1,062 |
| VC | -0,038 | 0,235 | 0,963 | 0,905 | 1,025 |
| IL-10 T0 | -0,382 | 0,38 | 0,682 | 0,291 | 1,602 |
| IL-10 T1 | -0,015 | 0,366 | 0,985 | 0,955 | 1,017 |
| IL-10 T2 | 0,002 | 0,993 | 1,002 | 0,598 | 1,679 |
| IL-8 T0 | -0,035 | 0,334 | 0,966 | 0,901 | 1,036 |
| IL-8 T1 | -0,002 | 0,864 | 0,998 | 0,977 | 1,02 |
| IL-8 T2 | -0,012 | 0,385 | 0,988 | 0,961 | 1,016 |
| IL-6 T0 | 0,064 | 0,053 | 1,066 | 0,999 | 1,138 |
| IL-6 T1 | -0,013 | 0,083 | 0,987 | 0,972 | 1,002 |
| IL-6 T2 | -0,007 | 0,266 | 0,993 | 0,981 | 1,005 |
| Nicotine | 0,263 | 0,792 | 1,301 | 0,183 | 9,248 |
| ARBs | -2,968 | 0,051 | 0,051 | 0,003 | 1,012 |
| surgical approach | -2,155 | **0,037** | **0,116** | 0,015 | 0,881 |
|  |  |  |  |  |  |

S4 Multivariate regression analysis of absolute interleukin levels. FEV_1_ = forced expiratory volume in 1 second; PEF = peak expiratory flow; VC = vital capacity; IL-10 = interleukin 10; IL-8 = interleukin 8; IL-6 = interleukin 6; T0 = Before surgery; T1 = at the end of surgery at wound closure; T2 = 24 hours after surgery; ARBs = Angiotensin-receptor-II blockers; surgical approach (thoracoscopy versus thoracotomy).
