# Supplementary material for: Perioperative cytokine profile during lung surgery predicts patients at risk for postoperative complications—A prospective, clinical study
Source: PLoS One. 2018 Jul 3;13(7):e0199807. doi: 10.1371/journal.pone.0199807 (PMC6029786; doi:10.1371/journal.pone.0199807)
Supplement: S3 Table — IL-10 = interleukin 10; IL-8 = interleukin 8; IL-6 = interleukin 6; T0 = Before surgery; T1 = at the end of surgery at wound closure; T2 = 24 hours after surgery. (DOCX) [file pone.0199807.s005.docx]

|  | Perzentile | | |
| --- | --- | --- | --- |
|  | 25. | 50. (Median) | 75. |
| IL-10 T0 | 0,00 | 0,00 | 1,00 |
| IL 10 T1 | 1,00 | 4,00 | 10,25 |
| IL-10 T2 | 0,00 | 1,00 | 2,00 |
| IL-8 T0 | 3,00 | 6,00 | 12,00 |
| IL-8 T1 | 11,00 | 17,00 | 25,00 |
| IL-8 T2 | 12,75 | 22,00 | 37,00 |
| IL-6 T0 | 0,00 | 1,00 | 8,00 |
| IL-6 T1 | 17,75 | 40,00 | 93,50 |
| IL-6 T2 | 35,75 | 70,00 | 110,25 |

S5 Aggregation of interleukin levels into quartiles. IL-10 = interleukin 10; IL-8 = interleukin 8; IL-6 = interleukin 6; T0 = Before surgery; T1 = at the end of surgery at wound closure; T2 = 24 hours after surgery
