# Supplementary material for: Perioperative cytokine profile during lung surgery predicts patients at risk for postoperative complications—A prospective, clinical study
Source: PLoS One. 2018 Jul 3;13(7):e0199807. doi: 10.1371/journal.pone.0199807 (PMC6029786; doi:10.1371/journal.pone.0199807)
Supplement: S4 Table — FEV1 = forced expiratory volume in 1 second; PEF = peak expiratory flow; VC = vital capacity; IL-6 = interleukin 6; T0 = Before surgery; T1 = at the end of surgery at wound closure; T2 = 24 hours after surgery; ARBs = Angiotensin-receptor-II blockers; surgical approach (thoracoscopy versus thoracotomy). (DOCX) [file pone.0199807.s006.docx]

|  | Regression coefficient | P Value | Odds Ratio Exp (B) | 95% Confidence interval | |
| --- | --- | --- | --- | --- | --- |
| FEV1 | 0,041 | 0,119 | 1,042 | 0,989 | 1,097 |
| PEF | -0,003 | 0,891 | 0,997 | 0,96 | 1,036 |
| VC | -0,019 | 0,45 | 0,981 | 0,935 | 1,03 |
| Nicotine | 0,297 | 0,741 | 1,346 | 0,23 | 7,867 |
| ARBs | -1,959 | 0,097 | 0,141 | 0,014 | 1,428 |
| surgical approach | -2,132 | **0,022** | **0,119** | 0,019 | 0,738 |
| IL-6 T1 <= 1. Quartile | 1,493 | 0,145 | 4,452 | 0,597 | 33,215 |
| IL-6 T1 >= 3. Quartile | 1,214 | 0,108 | 3,366 | 0,765 | 14,809 |
| IL-6 T2 <= 1. Quartile | -1,045 | 0,319 | 0,352 | 0,045 | 2,75 |
| IL-6 T2 >= 3. Quartile | 0,922 | 0,235 | 2,514 | 0,549 | 11,509 |

S6 Multivariate regression analysis for IL-6. FEV_1_ = forced expiratory volume in 1 second; PEF = peak expiratory flow; VC = vital capacity; IL-6 = interleukin 6; T0 = Before surgery; T1 = at the end of surgery at wound closure; T2 = 24 hours after surgery; ARBs = Angiotensin-receptor-II blockers; surgical approach (thoracoscopy versus thoracotomy).
