# Supplementary material for: Perioperative cytokine profile during lung surgery predicts patients at risk for postoperative complications—A prospective, clinical study
Source: PLoS One. 2018 Jul 3;13(7):e0199807. doi: 10.1371/journal.pone.0199807 (PMC6029786; doi:10.1371/journal.pone.0199807)
Supplement: S5 Table — FEV1 = forced expiratory volume in 1 second; PEF = peak expiratory flow; VC = vital capacity; IL-10 = interleukin 10; T0 = Before surgery; T1 = at the end of surgery at wound closure; T2 = 24 hours after surgery; ARBs = Angiotensin-receptor-II blockers; surgical approach (thoracoscopy versus thoracotomy). (DOCX) [file pone.0199807.s007.docx]

|  | Regression coefficient | P Value | Odds Ratio Exp (B) | 95% Confidence interval | |
| --- | --- | --- | --- | --- | --- |
| FEV1 | 0,02 | 0,429 | 1,021 | 0,97 | 1,073 |
| PEF | 0,009 | 0,585 | 1,009 | 0,976 | 1,044 |
| VC | -0,013 | 0,57 | 0,987 | 0,943 | 1,033 |
| Nicotine | 0,697 | 0,357 | 2,007 | 0,456 | 8,837 |
| ARBs | -2,118 | 0,072 | 0,12 | 0,012 | 1,206 |
| surgical approach | -2,233 | **0,014** | **0,107** | 0,018 | 0,633 |
| IL-10 T1 <= 1. Quartile | -1,161 | 0,14 | 0,313 | 0,067 | 1,461 |
| IL-10 T1 >= 3. Quartile | 0,113 | 0,893 | 1,12 | 0,216 | 5,796 |
| IL-10 T2 >= 3. Quartile | 0,088 | 0,896 | 1,092 | 0,291 | 4,093 |

S7 Multivariate regression analysis for IL-10. FEV_1_ = forced expiratory volume in 1 second; PEF = peak expiratory flow; VC = vital capacity; IL-10 = interleukin 10; T0 = Before surgery; T1 = at the end of surgery at wound closure; T2 = 24 hours after surgery; ARBs = Angiotensin-receptor-II blockers; surgical approach (thoracoscopy versus thoracotomy).
