# Supplementary material for: Perioperative cytokine profile during lung surgery predicts patients at risk for postoperative complications—A prospective, clinical study
Source: PLoS One. 2018 Jul 3;13(7):e0199807. doi: 10.1371/journal.pone.0199807 (PMC6029786; doi:10.1371/journal.pone.0199807)
Supplement: S6 Table — FEV1 = forced expiratory volume in 1 second; PEF = peak expiratory flow; VC = vital capacity; IL-6 = interleukin 6; IL-8 = interleukin 8; T1 = at the end of surgery at wound closure; ARBs = Angiotensin-receptor-II blockers; surgical approach (thoracoscopy versus thoracotomy. (DOCX) [file pone.0199807.s008.docx]

|  | Regression coefficient | P Value | Odds Ratio Exp (B) | 95% Confidence interval | |
| --- | --- | --- | --- | --- | --- |
| FEV1 | 0,018 | 0,394 | 1,019 | 0,976 | 1,063 |
| PEF | 0,02 | 0,21 | 1,02 | 0,989 | 1,053 |
| VC | -0,005 | 0,806 | 0,995 | 0,955 | 1,036 |
| Nicotine | 0,74 | 0,262 | 2,096 | 0,575 | 7,638 |
| ARBs | -2,089 | 0,063 | 0,124 | 0,014 | 1,124 |
| surgical approach | -2,256 | **0,003** | **0,105** | 0,024 | 0,466 |
| IL-6 T1 & IL-8 T1 >= 3. Quartile | 0,105 | 0,9 | 1,11 | 0,216 | 5,713 |

S8 Multivariate regression analysis for patients with IL-8 and IL-6 levels above the 3^rd^ quartile at the end of surgery. FEV_1_ = forced expiratory volume in 1 second; PEF = peak expiratory flow; VC = vital capacity; IL-6 = interleukin 6; IL-8 = interleukin 8; T1 = at the end of surgery at wound closure; ARBs = Angiotensin-receptor-II blockers; surgical approach (thoracoscopy versus thoracotomy
